# Genetic Variability of Leaf Mineral Concentrations in *Coffea canephora* Clones Cultivated in the Western Amazon

**DOI:** 10.3390/plants15152331

**Published:** 2026-07-29

**Authors:** Josemar Dávila Torres, Marcelo Curitiba Espindula, Rodrigo Barros Rocha, Alana Mara Kolln, Alexsandro Lara Teixeira, Larissa Fatarelli Bento de Araújo, Fábio Luiz Partelli

**Affiliations:** 1 Legal Amazon Biodiversity and Biotechnology Network (Bionorte), Federal University of Rondônia (UNIR), Porto Velho 76801-059, RO, Brazil; josemar-torres@hotmail.com (J.D.T.);; 2 Institute of Research, Technical Assistance and Rural Extension of Espírito Santo (INCAPER), Vitória 29052-010, ES, Brazil; 3 Embrapa Café, Brazilian Agricultural Research Corporation, Brasília 70770-901, DF, Brazil; 4 Federal Institute of Education, Science and Technology of Rondônia, Campus São Miguel do Guaporé, São Miguel do Guaporé 76932-000, RO, Brazil; 5 Embrapa Rondônia, Brazilian Agricultural Research Corporation, Porto Velho 76815-800, RO, Brazil; 6 Department of Agricultural and Biological Sciences, Northern Espírito Santo University Center, Federal University of Espírito Santo, São Mateus 29932-900, ES, Brazil

**Keywords:** Conilon, Robusta, genetic variability, mineral nutrition, heritability

## Abstract

Coffee cultivation in the Western Amazon is based on *Coffea canephora* Pierre ex A. Froehner clones with limited nutritional characterization. This study aimed to quantify the genetic variability in leaf mineral concentrations among 64 widely cultivated genotypes. The experiment was conducted at the Embrapa experimental field station in Ouro Preto do Oeste, Rondônia, using a randomized complete block design with three replications. Leaf concentrations of N, P, K, Ca, Mg, S, B, Cu, Fe, Mn, Zn, Na, and Al were evaluated. Significant genotype effects were observed for all evaluated elements (*p* ≤ 0.01), with high heritability estimates for K, Cu, Mn, N, and S. The decreasing order of leaf macronutrient concentrations was N > K > Ca > Mg > S > P. Among the evaluated elements, Fe, Mn, Zn, Na, and Al showed the greatest variation, with clones LB33, N8G8, BRS1216, and GJ30 standing out for their higher concentrations of Zn/Na, Al, N, and K, respectively. Multivariate analyses indicated that variation in foliar mineral concentrations was predominantly continuous, although the clones were partitioned into two groups with contrasting profiles. Group 1 was associated with higher concentrations of P, Cu, Fe, Mn, and Al, whereas Group 2 showed higher relative concentrations of N, K, Ca, Mg, S, Na, and Zn. These findings reveal distinct foliar mineral profiles among the evaluated clones and provide a basis for selecting contrasting genotypes for future studies on clone-specific nutritional responses.

## 1. Introduction

Coffee cultivation is an important agricultural activity in the Western Amazon, particularly in the state of Rondônia, which has become one of the leading Brazilian producers of *Coffea canephora* Pierre ex A. Froehner [1]. The expansion of this crop in the Amazon is associated with the high adaptability of the species to local soil and climatic conditions, characterized by a humid tropical climate, high temperatures, and predominantly acidic soils with low natural fertility [2,3]. The cultivation of clonal genotypes has played a key role in ensuring stability, uniformity, and productivity in coffee production systems [4,5].

The genetic basis of coffee cultivation in the Western Amazon was established through the introduction of genotypes from the Conilon and Robusta botanical varieties [6]. The Conilon group was introduced mainly by migrant farmers from the state of Espírito Santo, whereas the Robusta group originated from materials introduced by research institutions, forming populations characterized by greater vigor and larger bean size [1]. Over time, recombination between these groups resulted in highly heterogeneous populations, increasing the genetic variability available in regional production systems [7].

Most genotypes currently cultivated exhibit characteristics derived from these two botanical varieties. In general, the Conilon group is associated with earlier maturation, shorter plant stature, and greater drought tolerance, whereas the Robusta group tends to exhibit greater vegetative vigor and larger bean size [8,9]. Recombination between these groups is a fundamental aspect of the genetics of cultivated clones, contributing to the expression of complementary traits between Conilon and Robusta materials [10]. This broad genetic background may also contribute to differences in mineral uptake, transport, and partitioning among genotypes, resulting in contrasting foliar mineral profiles.

In this context, particular attention should be given to farmer-selected clones among the most widely cultivated materials. These genotypes were empirically selected over decades from superior plants identified under field conditions, without formal pedigree control. Several of these materials were extensively disseminated across farms and regions, giving rise to widely cultivated clones such as GJ8, GJ25, and AS2. Despite their high agronomic importance and widespread adoption, these clones remain poorly characterized regarding physiological and nutritional aspects, highlighting an important knowledge gap concerning the magnitude and structure of variation in their leaf mineral concentrations.

At the same time, breeding programs have contributed to expanding the technological development of coffee cultivation. In this regard, the Brazilian Agricultural Research Corporation (Embrapa) released ten clonal cultivars adapted to the conditions of the Western Amazon in 2019, aiming to complement the set of materials cultivated in the region [3]. Among these materials, BRS1216 has gained increasing adoption in commercial plantations, illustrating the practical relevance of improved clones in regional production systems. These genotypes broaden the spectrum of available genetic variability and allow comparisons between farmer-selected clones and materials developed through formal breeding programs.

Mineral nutrition is one of the main factors determining coffee plant growth, productivity, and longevity [11,12]. The analysis of nutrient concentrations in plant tissues is widely used as a nutritional diagnostic tool, allowing the identification of nutritional limitations and the adjustment of fertilization recommendations [13,14]. Genotypic variation in leaf mineral concentrations may reveal contrasting nutritional profiles and help identify materials of interest for subsequent studies on nutrient uptake and use, contributing to the development of more efficient and sustainable fertilization strategies [15,16]. In addition to essential nutrients, the evaluation of Na and Al may provide complementary information on ionic balance and mineral responses under tropical soil conditions.

The extent to which the most widely cultivated clones in the Western Amazon differ in their foliar mineral profiles remains poorly understood, particularly when farmer-selected materials and improved cultivars are evaluated together. We hypothesized that the evaluated clones exhibit significant genotypic variation and contrasting multivariate patterns in leaf mineral concentrations. Therefore, this study aimed to quantify the genotypic variability in leaf mineral concentrations among *C. canephora* clones cultivated in the Western Amazon and to characterize the associations and multivariate patterns among the evaluated elements, providing a basis for identifying contrasting genotypes for future nutritional and breeding studies.

## 2. Results and Discussion

### 2.1. Genetic Variability of Leaf Nutrient Concentrations

Analysis of variance revealed significant genetic differences (*p* < 0.01) among the 64 *C. canephora* clones for all evaluated leaf nutrients (Table 1), demonstrating broad nutritional variability within the studied population. Similar results have been reported under different soil and climatic conditions [17,18], confirming the existence of genetic diversity regarding nutrient accumulation in this coffee species.

Broad-sense heritability estimates (h^2^) were high for most elements, ranging from 51.48% for boron to 81.63% for copper. Particularly high values were observed for potassium (81.06%), copper (81.63%), manganese (74.22%), nitrogen (66.23%), and sulfur (66.11%), indicating a greater contribution of the genetic component to the phenotypic variation under the evaluated conditions. In clonal *C. canephora* cultivars developed for the Western Amazon, Starling et al. [19] also reported high heritability estimates for leaf macronutrients, reinforcing the importance of genotype in the expression of these traits.

Experimental coefficients of variation (CVe) showed low to moderate magnitudes for most elements, indicating adequate experimental precision. Values lower than 20% were observed for most variables, according to the criteria described by Giles et al. [20]. Exceptions were sulfur (34.01%), sodium (34.14%), and zinc (105.85%), which showed greater residual variation relative to their respective means. In the case of zinc, the high CVe may be associated with the low concentration of this micronutrient in leaf tissues, which increases the relative effect of residual variation on the coefficient, as commonly observed for micronutrients with low concentrations [21,22].

The CVg/CVe ratio was higher than unity for potassium (1.19) and copper (1.22), indicating a greater magnitude of genotypic variation relative to residual variation for these elements under the evaluated conditions. For the remaining elements, values ranged from 0.59 to 0.98, indicating lower but still detectable genotypic variation relative to the experimental variation. These results highlight the possibility of exploiting this variability in breeding programs aimed at differentiating clones according to their leaf mineral profiles.

### 2.2. Macronutrient Accumulation Patterns

Mean macronutrient concentrations exhibited substantial variation among the 64 evaluated genotypes (Table 2), evidencing genetic differences in leaf macronutrient profiles. Similar variation has been reported by Gomes et al. [17] and Martins et al. [23], indicating that leaf nutrient concentrations may differ substantially among clones cultivated under tropical conditions.

The decreasing order of mean leaf macronutrient concentrations was N > K > Ca > Mg > S > P. This pattern is similar to that reported for clonal cultivars developed for the Brazilian Amazon [24], reinforcing the predominance of nitrogen and potassium in the leaf mineral profile of the evaluated clones. However, differences in the relative concentrations of these elements have been reported in other growing regions, especially under higher-altitude conditions or different management systems [25], highlighting the combined influence of genetic and environmental factors on coffee nutritional dynamics [26].

Among the macronutrients, nitrogen showed substantial variation, with concentrations ranging from 16.29 to 30.42 g kg^−1^. The highest values were observed in clones BRS1216, VP156, and R152, whereas clone SK80 exhibited the lowest leaf concentration. Potassium also showed substantial variability, ranging from 11.75 to 20.13 g kg^−1^, with emphasis on clones GJ30, GJ25, and GJ8. The high variability observed for N and K is consistent with the elevated heritability estimates and indicates marked genotypic differences in the leaf concentrations of these elements under the evaluated conditions [27].

Phosphorus concentrations ranged from 0.60 to 0.98 g kg^−1^, forming only two statistical groups and indicating lower variability among genotypes. This behavior suggests a comparatively narrower range of leaf phosphorus concentrations among the evaluated clones. Despite this lower univariate variation, phosphorus contributed substantially to the multivariate differentiation of the genotypes and showed significant associations with copper, iron, manganese, and zinc, indicating that its relevance became more evident when considered jointly with the other elements. In contrast, calcium and magnesium showed moderate variation among clones, with the highest concentrations observed in AS7 and AS10. The association between these nutrients was also confirmed by the correlation analysis, indicating coordinated variation in their leaf concentrations. Their similar behavior also contributed to the distinction of foliar mineral profiles among the evaluated clones.

Sulfur concentrations ranged from 0.42 to 2.25 g kg^−1^, with RE22 and GJ8 showing the highest numerical means. Despite the observed amplitude, sulfur exhibited a relatively high experimental coefficient of variation, indicating greater residual variation relative to the mean concentration of this element [28]. Overall, the differences observed among clones for the evaluated macronutrients suggest the existence of partially distinct leaf mineral profiles under the evaluated conditions [23].

### 2.3. Micronutrient, Sodium, and Aluminum Accumulation Patterns

Leaf concentrations of micronutrients, sodium, and aluminum varied among the evaluated clones, demonstrating differences in foliar mineral profiles (Table 3). Although some elements exhibited relatively homogeneous concentrations, others showed greater variation among genotypes.

Boron concentrations ranged from 62.05 to 92.64 mg kg^−1^, with the highest values observed in clones RE22 and N14. Despite the relatively moderate amplitude, concentrations remained within the range commonly reported for coffee plants cultivated under tropical conditions, as described by Ramirez-Builes et al. [29] and Santinato et al. [30]. Although boron showed the lowest broad-sense heritability estimate among the evaluated elements, the significant genotypic variation and its contribution to the multivariate structure indicate that this element helped differentiate the foliar mineral profiles of the clones. Iron concentrations also varied among genotypes, with LB88 exhibiting the highest mean value. Iron was among the elements that contributed most strongly to the multivariate differentiation, particularly characterizing the group associated with higher relative concentrations of P, Cu, Fe, Mn, and Al.

Among the micronutrients, iron, manganese, and zinc exhibited the greatest variation among clones. For zinc, clone LB33 stood out remarkably, reaching 110.00 mg kg^−1^, substantially higher than the concentrations observed in the other evaluated materials. Differences in leaf concentrations of these nutrients may reflect effects related to vegetative growth, internal redistribution, and variation in leaf expansion rate, producing dilution or concentration effects within plant tissues.

Sodium concentrations exhibited variation among clones, ranging from 23.00 to 74.50 mg kg^−1^. The highest concentrations were observed in clones LB33 and AR106. Although sodium is not considered an essential element for coffee and no saline conditions were identified in the experimental area, its variation among clones contributed to the differentiation of their foliar mineral profiles. These results indicate genotypic variation in leaf Na concentration under the same cultivation conditions, although the physiological processes underlying these differences were not evaluated. Aluminum concentrations ranged from 165.00 to 302.50 mg kg^−1^, with clone N8G8 exhibiting the highest value. The soil analysis indicated no exchangeable Al at the sampled depths, despite its acidic characteristics and the need for liming before planting. Thus, the variation in leaf Al concentration occurred under conditions of low apparent Al availability in the soil and may reflect differences among genotypes in the amount of this element reaching the leaves. However, leaf concentration alone does not allow conclusions regarding Al uptake, compartmentalization, or tolerance. Both Na and Al contributed to the multivariate differentiation of the evaluated clones [31,32].

Overall, the results demonstrate that the nutritional variability observed for micronutrients does not occur uniformly among the evaluated elements. Whereas some nutrients exhibited relatively stable behavior among clones, others—especially Fe, Mn, Zn, Na, and Al—contributed strongly to nutritional differentiation among genotypes, suggesting a possible role for these elements in the multivariate structure later observed in the principal component analyses.

### 2.4. Interactions in Nutrient Concentrations

**Figure 1:**
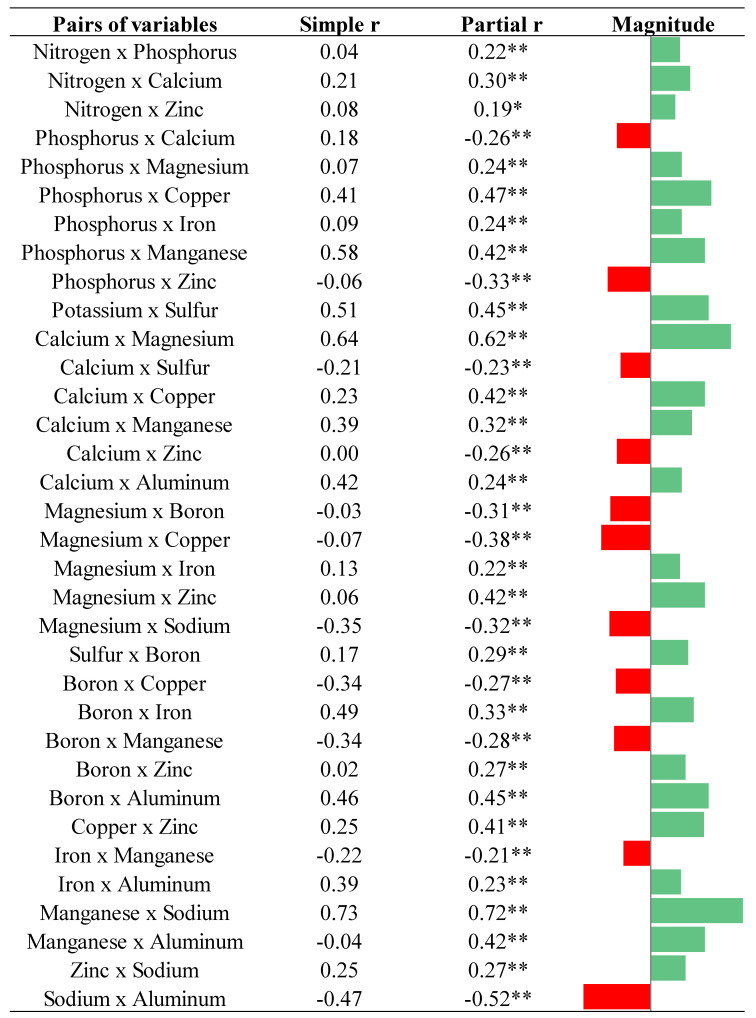
Significant estimates of simple and partial correlations among foliar mineral concentrations of 64 clones evaluated in a clonal competition trial conducted at the Embrapa experimental field in Ouro Preto do Oeste, Rondônia, Brazil. Green bars indicate positive partial correlations, whereas red bars indicate negative partial correlations. Bar length represents the absolute magnitude of the partial correlation coefficient. *: significant at the 5% probability level; **: significant at the 1% probability level.

Partial correlation analysis allowed the identification of associations among leaf nutrient concentrations while controlling for the effects of the remaining elements (Figure 1). This approach was useful for distinguishing conditional associations from those observed in the simple correlation matrix. In total, 34 significant partial correlations were identified, of which 11 were negative.

Among the strongest positive associations were the correlations between calcium and magnesium (r = 0.62 **), manganese and sodium (r = 0.72 **), and potassium and sulfur (r = 0.45 **), where ** indicates significance at the 1% probability level. The strong association between calcium and magnesium indicates coordinated variation in the leaf concentrations of these divalent cations, which may be related to shared physiological processes involving nutrient transport and redistribution within the plant. Similarly, the association between potassium and sulfur indicates that clones with higher leaf potassium concentrations also tended to exhibit higher sulfur concentrations [33].

The strong positive correlation between manganese and sodium was one of the most expressive associations observed in this study, indicating simultaneous variation in the leaf concentrations of these elements among clones. Positive correlations were also observed between phosphorus and copper (r = 0.47 **), phosphorus and iron (r = 0.24 **), and magnesium and zinc (r = 0.42 **), showing that the concentrations of these pairs of elements varied in the same direction among the evaluated genotypes.

Conversely, notable negative correlations were observed between phosphorus and zinc (r = −0.33 **) and sodium and aluminum (r = −0.52 **). These results indicate that higher leaf concentrations of one element were associated with lower concentrations of the other within each pair, without necessarily implying a direct physiological antagonism. Negative correlations were also observed between magnesium and boron (r = −0.31 **) and calcium and zinc (r = −0.26 **). Additional positive associations included phosphorus and manganese (r = 0.42 **), reinforcing the coordinated variation among some elements in the leaf mineral profiles of the clones.

Overall, the simultaneous occurrence of positive and negative associations indicates that the elements did not vary independently across genotypes. These relationships contributed to the differentiation of leaf mineral profiles among the evaluated clones [34,35,36].

### 2.5. Genotypic Variation in Leaf Mineral Profiles

These results indicate that variation in leaf mineral concentrations may be associated with the genetic background present in the studied population. The historical introduction of materials belonging to the Conilon and Robusta botanical varieties, followed by natural hybridization and selection carried out by farmers and breeding programs, contributed to the formation of populations with hybrid characteristics [6,7,37].

Genotypes with a greater genetic contribution from the Conilon variety tend to exhibit earlier maturation and shorter plant stature, whereas materials with stronger Robusta influence frequently display greater vigor and vegetative development [9]. Although the relationship between genetic background and leaf mineral concentrations was not directly tested in this study, some clones exhibited contrasting leaf nutrient profiles. Materials belonging to the GJ group, especially GJ30, GJ25, GJ8, and GJ2, stood out for their high leaf potassium concentrations, frequently associated with elevated sulfur concentrations. In contrast, clones AS7 and AS10 exhibited higher calcium and magnesium concentrations, indicating similar leaf mineral profiles for these elements.

Among the micronutrients, some genotypes exhibited particularly distinct profiles. Clone LB33 stood out for the simultaneous high leaf concentrations of zinc and sodium, representing one of the most contrasting nutritional profiles within the experimental set. Clone LB88 exhibited the highest iron concentration, whereas SK88 and GB4 stood out for their elevated manganese concentrations. Similarly, BRS1216 exhibited a high leaf nitrogen concentration, whereas SK80 showed the opposite behavior, with a lower concentration of this element.

The results obtained in this study demonstrate that variation in leaf mineral concentrations among clones occurred predominantly in a continuous manner, without abrupt separation among the evaluated genotypes. This behavior is compatible with the hybrid nature of cultivated populations, in which characteristics associated with the Conilon and Robusta botanical groups are extensively integrated [10,36,38].

### 2.6. Multivariate Structure and Nutritional Variability

**Figure 2:**
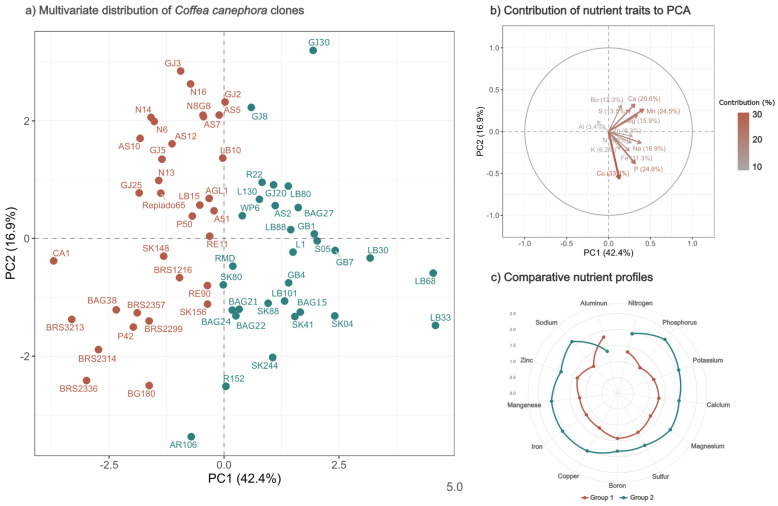
Multivariate analysis of leaf mineral variability among 64 *Coffea canephora* clones cultivated in the Western Amazon. (**a**) Principal component analysis showing the spatial distribution of clones and their classification into two contrasting profiles using k-means clustering. (**b**) Correlation circle representing the contribution of macro- and micronutrients to the first two principal components (PC1 and PC2). (**c**) Radar chart showing the standardized nutrient profiles of the two groups obtained by k-means clustering. The analyses were based on the foliar concentrations of N, P, K, Ca, Mg, S, B, Cu, Fe, Mn, Zn, Na, and Al.

Principal component analysis (PCA) allowed the integration of the variability observed among the different leaf nutrients and enabled the identification of multivariate nutrient concentration patterns among the evaluated *C. canephora* clones (Figure 2). The first two principal components jointly explained 59.3% of the total data variability, with 42.4% associated with the first component (PC1) and 16.9% with the second component (PC2). The distribution of genotypes along these components revealed the main gradients of variation in leaf mineral concentrations, which were subsequently summarized into two contrasting nutritional profiles.

The nutrients that contributed most strongly to the multivariate structure were phosphorus, copper, iron, manganese, calcium, sodium, and boron, reflecting the greater discriminatory capacity of these elements among the evaluated clones. In contrast, nutrients such as aluminum, potassium, and zinc showed a lower relative contribution to principal component formation, although they contributed to the differentiation of specific genotypes. Vector orientation revealed positive associations among calcium, magnesium, manganese, and boron, whereas phosphorus, copper, and iron were associated with genotypes grouped in the left quadrants of the ordination (Figure 2).

The distribution of genotypes along PC1 revealed two main nutritional patterns. Clones positioned on the right side of the ordination were associated with higher relative concentrations of nitrogen, potassium, calcium, magnesium, sodium, and zinc, including materials such as GJ30, GJ8, LB33, LB68, LB30, SK41, and BAG15. Among these materials, GJ30 stood out for its elevated potassium concentration, whereas LB33 showed a strong association with zinc and sodium, occupying one of the most extreme positions in the multivariate ordination. Clones AS2, R22, BAG27, and GB1 were also positioned close to this region of the PCA, indicating greater similarity in leaf mineral profiles to the materials associated with the group of variables represented by N, K, Ca, and Mg.

In contrast, genotypes positioned on the left side of the ordination showed greater association with phosphorus, copper, iron, manganese, and aluminum, particularly clones RE22, N14, AS10, BRS3213, BRS2336, BAG38, BG180, and P42. Clones AS7, AS12, and N8G8 also exhibited relatively close distribution within this region, especially in association with calcium, magnesium, and boron. Some materials, such as CA1, AR106, and SK244, exhibited more isolated positions in the multivariate space, contributing to the broader dispersion observed among the evaluated genotypes.

K-means clustering applied to the scores of PC1 and PC2, with k = 2 defined a priori, partitioned the clones into two groups with contrasting mean leaf mineral profiles. The first group concentrated genotypes associated with higher relative concentrations of phosphorus, copper, iron, manganese, and aluminum, whereas the second group showed a tendency toward greater concentrations of nitrogen, potassium, calcium, magnesium, sulfur, sodium, and zinc. The mean profiles obtained from the radar plots showed high agreement with the gradients observed in the PCA, indicating that the two-group solution provided a useful summary of the main patterns of variation in the studied population.

Although the evaluated elements did not contribute equally to the principal components, their joint variation distinguished two contrasting foliar mineral profiles. Multivariate approaches have been used to integrate nutrient relationships and support the development of nutritional diagnostic frameworks [12,13,14].

These contrasting profiles provide a practical framework for selecting representative clones and for future studies evaluating group-specific nutritional diagnosis. They also indicate that the adequacy of using a single nutritional reference for all clones should be further evaluated before the development and validation of group-specific DRIS norms.

## 3. Materials and Methods

### 3.1. Field Experiment

The experiment was conducted at the Embrapa Experimental Field Station in Ouro Preto do Oeste, Rondônia, Brazil (10°43′55″ S, 62°15′19″ W; altitude 300 m). The regional climate is classified as tropical monsoon (Am) according to the Köppen classification system, with a mean annual temperature of 25 °C and annual rainfall of approximately 2000 mm [39].

The soil of the experimental area is classified as a dystrophic Yellow–Red Oxisol. The area was cultivated with *C. canephora* coffee plants between 2011 and 2018 and subsequently remained fallow until the establishment of the experiment. Before the experiment was established, soil chemical properties were evaluated at depths of 0–10 cm and 10–20 cm during field preparation (Table 4).

Before planting, lime was applied at 1400 kg ha^−1^ to increase base saturation to 70% and incorporated by plowing and harrowing to a depth of 20 cm. After soil preparation, furrows 80 cm deep were opened, and 3 kg of poultry litter per linear meter was incorporated before transplanting the seedlings.

The experiment was established on 24 and 25 February 2022, using a spacing of 3.0 m between rows and 0.75 m between plants. Clonal seedlings approximately 120 days old were produced in the nursery of the experimental field station.

On 27 April 2022, seeds of Crotalaria spectabilis were sown between the rows at a density of 20 kg ha^−1^. The plants were cut in July 2022, and the residues were maintained on the soil surface as mulch.

In August 2022, apical pruning was performed to standardize the number of stems per plant [40]. In October 2022, 90 days after pruning, new shoots were selected, maintaining two orthotropic stems per plant.

During the establishment phase, from April 2022 to July 2023, 160 kg ha^−1^ of N, 50 kg ha^−1^ of P_2_ O_5_, and 60 kg ha^−1^ of K_2_ O were applied using ammonium sulfate, urea, monoammonium phosphate (MAP), and a 20-05-20 fertilizer formulation. Additionally, 40 kg ha^−1^ of magnesium sulfate, 8 kg ha^−1^ of zinc sulfate, and 4 kg ha^−1^ of borax were applied.

Between August and November 2023, topdressing fertilization consisted of 30 g plant^−1^ of a 20-05-20 fertilizer formulation, 50 g plant^−1^ of urea, and 30 g plant^−1^ of FTE BR-13 micronutrient fertilizer containing 7.0% Zn, 1.8% B, 2.0% Cu, 2.0% Fe, and 0.1% Mo.

The experiment was conducted in a randomized complete block design with three replications, with each plot consisting of five plants.

### 3.2. Genetic Material

The study comprised 64 *C. canephora* genotypes representative of the genetic variability currently cultivated in the Western Amazon (Table 5). The experimental set consisted of 52 public-domain clones, six cultivars, and six breeding genotypes developed by Embrapa and currently under agronomic evaluation [41,42]. Detailed genetic characterization of these clones, including SNP-based analyses, DAPC, and estimates of Conilon and Robusta ancestry, is available in Rocha et al. (2025) [10].

The evaluated materials included clones empirically selected by pioneer coffee growers in the region, as well as genotypes derived from breeding programs. Among the locally selected clones, materials selected by farmers such as Geraldo Jacomin, Laerte Braun, and Sérgio Kalk stand out, as they are widely disseminated throughout regional production systems.

The collection also included cultivars released by Embrapa, identified by the prefix “BRS”, such as BRS1216, BRS2314, BRS2357, and BRS3213, in addition to genotypes belonging to pre-breeding programs maintained in active germplasm banks (BAG15, BAG21, BAG22, BAG24, BAG27, and BAG38).

The genetic background of the evaluated materials is predominantly composed of hybrid genotypes derived from the Conilon and Robusta botanical varieties, characterized by high adaptation to the soil and climatic conditions of the Western Amazon.

### 3.3. Leaf Analyses

To determine leaf mineral concentrations, four leaves were collected per plant from the five sampled plants within each plot, totaling 20 leaves per composite sample. Sampling was performed in November 2023, during the fruit expansion stage, following recommendations for coffee leaf nutritional diagnosis [43,44].

In each experimental plot, one composite sample consisting of 20 fully expanded leaves was collected. Leaves were sampled from plagiotropic branches located in the middle third of the plant canopy, from the third or fourth pair of leaves from the branch apex. Sampling was performed in a standardized manner by collecting leaves from plants distributed across the four cardinal directions of the plot to adequately represent plant nutritional status and minimize variation associated with leaf position within the canopy. The composite sample was considered the experimental unit for the laboratory and statistical analyses.

The leaves were placed in paper bags, identified, and transported in expanded polystyrene containers to the Soil Laboratory of Embrapa Rondônia. In the laboratory, the samples were dried in a forced-air circulation oven at 65 °C until constant weight. Subsequently, the material was ground using a Wiley mill to obtain homogeneous samples.

Concentrations of nitrogen (N), phosphorus (P), potassium (K), calcium (Ca), magnesium (Mg), sulfur (S), boron (B), copper (Cu), iron (Fe), manganese (Mn), zinc (Zn), sodium (Na), and aluminum (Al) were determined according to the methodology described by Malavolta, Vitti, and Oliveira [43]. Macronutrient concentrations were expressed as g kg^−1^ dry matter, whereas micronutrient, Na, and Al concentrations were expressed as mg kg^−1^ dry matter. Chlorine and nickel were not included because they were not part of the analytical protocol routinely adopted by the laboratory for coffee leaf analysis.

### 3.4. Statistical Analyses

The data were initially inspected for outliers and the residuals were evaluated for normality and homogeneity of variances. Normality was assessed through graphical inspection of residuals and the Shapiro–Wilk test [45].

Data were subjected to analysis of variance (ANOVA) considering a randomized complete block design with three replications, according to the following linear model:

$$Y_{ij}=\mu +B_{j}+G_{i}+e_{ij}$$

where *Y_ij_* represents the observation of the *i*-th genotype in the *j*-th block, *μ* is the overall mean, *B_j_* is the block effect, *G_i_* is the genotype effect, and *e_ij_* is the random experimental error. Block and genotype effects were considered random.

The significance of genotype effects was tested according to the procedures described by Cruz [45]. Genetic parameters were estimated from the variance components obtained in the analysis of variance, including broad-sense heritability on a genotype-mean basis (h^2^), the genetic coefficient of variation (*CV_g_*), the experimental coefficient of variation (*CV_e_*), and the *CV_g_*/*CV_e_* ratio. Broad-sense heritability was estimated as:

$$h^{2}=\frac{\sigma_{g}^{2}}{\sigma_{g}^{2}+\sigma_{e}^{2}/r}$$

where $\sigma_{g}^{2}$ is the genotypic variance, $\sigma_{e}^{2}$ is the residual variance, and *r* is the number of replications. The genetic and experimental coefficients of variation were calculated as

$${CV}_{g}=\frac{\sqrt{\sigma_{g}^{2}}}{\bar{x}}$$

and

$${CV}_{e}=\frac{\sqrt{\sigma_{e}^{2}}}{\bar{x}}$$

respectively, where $\bar{x}$ is the overall mean. The *CV_g_*/*CV_e_* ratio was used to describe the relative magnitude of genotypic and residual variation under the evaluated conditions.

Genotype means were compared using the Scott–Knott test (*p* ≤ 0.05) implemented in the Genes software [45]. Associations among leaf mineral concentrations were evaluated using the means of the 64 genotypes and Pearson’s correlation coefficient (r). Partial correlation analysis was also performed to assess associations between pairs of elements while statistically controlling for the effects of the remaining variables. The significance of the coefficients was tested at the 5% and 1% probability levels.

Principal component analysis (PCA) was performed using the mean mineral concentrations of the 64 genotypes, standardized to have a mean of zero and unit variance, thereby eliminating the effect of differences in measurement scales among variables. The analysis was based on the correlation matrix, and the principal components were obtained from its eigenvalues and eigenvectors. Eigenvalues were used to quantify the variance explained by each component, whereas eigenvectors (loadings) indicated the contribution of each variable to component formation. Analyses were performed in the R statistical environment, version 4.5.0 [46], using the FactoMineR package for PCA, the factoextra package for extraction and visualization of principal components, and the ggplot2 package for graphical visualization.

The first two principal components were retained because they accounted for the largest proportion of the total variance and represented the main gradients of variation among genotypes and elements. The results were visualized using a biplot showing genotype distribution and variable contributions. K-means clustering was subsequently applied to the scores of the first two principal components, with k = 2 defined a priori to provide a practical summary of the main contrast in leaf mineral profiles rather than to determine the natural number of groups in the population. For each group, the standardized mean concentrations of the 13 evaluated elements were calculated and displayed in a radar chart.

## 4. Conclusions

*C. canephora* clones cultivated in the Western Amazon exhibited wide genetic variability in leaf macro- and micronutrient concentrations, as evidenced by significant differences among genotypes and high broad-sense heritability estimates for elements such as potassium, copper, manganese, nitrogen, and sulfur. Among the micronutrients and other evaluated elements, iron, manganese, zinc, sodium, and aluminum showed broad variation among clones, indicating marked differences in leaf mineral profiles under the evaluated conditions. Multivariate analyses demonstrated that this variability occurred predominantly in a continuous manner, although k-means clustering partitioned the clones into two groups with contrasting mean foliar mineral profiles. The first group showed greater association with phosphorus, copper, iron, manganese, and aluminum, whereas the second group exhibited higher relative concentrations of nitrogen, potassium, calcium, magnesium, sulfur, sodium, and zinc. Clones such as LB33, N8G8, BRS1216, and GJ30 stood out for their high leaf concentrations of Zn and Na, Al, N, and K, respectively, highlighting relevant contrasts among the materials cultivated in the region. The identification of these contrasting foliar mineral profiles provides useful information for selecting genotypes for breeding and nutritional studies and establishes a preliminary basis for the future development and validation of group-specific DRIS norms for *C. canephora*.

## Figures and Tables

**Table 1:** Estimates of genetic parameters and mean squares for leaf mineral concentrations in 64 *Coffea canephora* clones evaluated in the competition trial conducted at the Embrapa experimental field in Ouro Preto do Oeste, Rondônia, Brazil.

SV	DF	Nitrogen	Phosphorus	Potassium	Calcium	Magnesium	Sulfur	Boron
Blocks	2	52.01	0.04	98.13	0.74	6.11	0.01	5765.74
Genotypes	63	16.90 **	0.0147 **	10.56 **	2.99 **	0.17 **	0.45 **	144.51 **
Residual	126	5.71	0.01	2.00	1.20	0.06	0.15	70.11
Mean		22.50	0.75	15.24	12.53	2.93	1.15	77.17
CVe		10.62	10.38	9.28	8.73	8.41	34.01	10.85
h^2^		66.23	58.52	81.06	59.96	63.19	66.11	51.48
CVg/CVe		0.81	0.69	1.19	0.71	0.76	0.81	0.59
**SV**	**DF**	**Copper**	**Iron**	**Manganese**	**Zinc**	**Sodium**	**Aluminum**	
Blocks	2	20.82	2990.72	2594.63	146.26	9030.94	1675.88	
Genotypes	63	4.03 **	2001.07 **	942.63 **	492.33 **	612.67 **	3022.51 **	
Residual	126	0.74	925.64	243.04	169.11	246.65	1247.31	
Mean		5.78	201.45	124.02	12.29	46.00	226.45	
CVe		14.89	15.10	12.57	105.85	34.14	15.60	
h^2^		81.63	53.74	74.22	65.65	59.74	58.73	
CVg/CVe		1.22	0.62	0.98	0.8	0.7	0.69	

** Significant at the 1% probability level by the F test.; SV: source of variation; DF: degrees of freedom; CVe: experimental coefficient of variation; h^2^, broad-sense heritability (%); CVg/CVe, ratio between the genetic and experimental coefficients of variation. Concentrations of N, P, K, Ca, Mg, and S are expressed in g kg^−1^, whereas concentrations of B, Cu, Fe, Mn, Zn, Na, and Al are expressed in mg kg^−1^ dry matter.

**Table 2:** Mean macronutrient concentrations of the 64 clones evaluated in the clonal competition trial conducted in the municipality of Ouro Preto do Oeste, Rondônia, Brazil.

Clone	Nitrogen	Phosphorus	Potassium	Calcium	Magnesium	Sulfur
AR106	19.03 c	0.81 a	15.37 b	11.62 b	2.35 b	1.14 b
R152	27.34 a	0.84 a	17.12 b	13.62 a	2.79 b	1.06 b
BG180	18.09 c	0.85 a	16.75 b	12.37 b	2.56 b	1.14 b
P42	20.31 c	0.82 a	13.62 c	11.87 b	2.75 b	0.85 b
CA1	21.39 c	0.78 a	16.50 b	12.62 a	2.98 a	1.03 b
RMD	22.48 b	0.80 a	15.50 b	12.00 b	3.17 a	1.03 b
GB7	25.64 a	0.91 a	13.62 c	12.87 a	3.10 a	1.38 a
R22	22.76 b	0.89 a	16.25 b	12.62 a	2.96 a	1.21 b
S05	24.79 b	0.89 a	16.50 b	12.37 b	2.94 a	1.39 a
WP6	23.28 b	0.82 a	15.37 b	13.12 a	3.06 a	1.42 a
L130	22.95 b	0.77 a	17.25 b	13.75 a	3.10 a	1.28 a
L1	23.00 b	0.82 a	16.75 b	12.50 a	3.06 a	1.56 a
GB1	21.44 c	0.71 b	15.00 c	13.25 a	3.11 a	1.10 b
GB4	21.07 c	0.98 a	16.00 b	13.25 a	2.91 a	1.03 b
AGL1	21.35 c	0.66 b	13.50 c	12.50 a	2.76 b	1.06 b
RE11	20.64 c	0.72 b	15.75 b	13.00 a	2.69 b	0.89 b
RE90	19.60 c	0.60 b	12.50 c	10.50 b	2.57 b	0.88 b
SK04	17.48 c	0.82 a	16.12 b	13.75 a	3.20 a	0.96 b
SK41	20.07 c	0.78 a	15.37 b	13.37 a	3.01 a	1.46 a
SK80	16.29 c	0.74 b	17.12 b	12.12 b	2.94 a	1.35 a
SK88	25.36 a	0.76 b	15.75 b	13.37 a	2.80 b	1.28 a
SK244	24.56 b	0.74 b	14.87 c	13.25 a	2.96 a	1.60 a
VP156	27.44 a	0.79 a	15.62 b	13.62 a	2.75 b	1.13 b
VP148	24.13 b	0.77 a	13.75 c	14.00 a	2.96 a	0.67 b
P50	22.01 c	0.72 b	14.37 c	14.12 a	3.25 a	0.78 b
Repiado	21.53 c	0.69 b	12.37 c	12.62 a	2.96 a	0.42 b
AS1	23.61 b	0.71 b	13.25 c	12.75 a	3.10 a	0.56 b
AS2	23.90 b	0.71 b	11.75 c	12.75 a	3.14 a	0.46 b
AS5	22.77 b	0.75 b	12.12 c	13.50 a	3.26 a	1.07 b
AS7	23.33 b	0.73 b	14.37 c	15.00 a	3.51 a	0.53 b
AS10	21.21 c	0.72 b	13.87 c	14.37 a	3.47 a	0.85 b
AS12	25.83 a	0.79 a	13.87 c	13.87 a	3.34 a	0.99 b
N13	23.61 b	0.69 b	13.25 c	12.87 a	2.93 a	0.96 b
N14	21.01 c	0.71 b	12.00 c	13.25 a	2.80 b	0.99 b
N16	22.95 b	0.69 b	11.75 c	12.25 b	2.90 a	1.20 b
N6	22.05 c	0.71 b	14.62 c	13.62 a	3.11 a	0.99 b
N8G8	21.82 c	0.73 b	17.12 b	14.12 a	3.40 a	1.06 b
RE22	22.86 b	0.69 b	17.75 a	12.75 a	2.89 b	2.25 a
GJ5	21.96 c	0.65 b	16.50 b	12.62 a	2.89 b	1.31 a
GJ8	21.91 c	0.79 a	18.62 a	12.75 a	3.10 a	2.18 a
GJ25	20.97 c	0.68 b	19.00 a	12.00 b	2.96 a	0.63 b
GJ2	23.05 b	0.68 b	18.62 a	11.87 b	3.05 a	1.85 a
GJ20	22.53 b	0.75 b	16.87 b	11.37 b	3.05 a	1.64 a
GJ30	21.35 c	0.72 b	20.13 a	11.87 b	2.97 a	1.82 a
LB10	21.25 c	0.65 b	13.50 c	11.25 b	2.59 b	1.39 a
LB15	21.25 c	0.74 b	16.25 b	11.37 b	2.75 b	1.75 a
LB30	22.01 c	0.67 b	17.87 a	11.87 b	2.95 a	1.53 a
LB33	23.23 b	0.70 b	14.62 c	12.50 a	2.99 a	1.21 b
LB68	24.08 b	0.86 a	14.62 c	11.50 b	2.89 b	1.81 a
LB80	22.72 b	0.72 b	14.37 c	11.00 b	2.65 b	1.49 a
LB88	22.48 b	0.68 b	16.00 b	11.75 b	2.74 b	0.56 b
LB101	23.05 b	0.72 b	15.37 b	12.12 b	2.71 b	0.99 b
BAG15	22.48 b	0.78 a	18.37 a	10.87 b	2.90 a	1.06 b
BAG21	23.10 b	0.70 b	13.75 c	11.00 b	2.69 b	0.70 b
BAG22	22.90 b	0.70 b	15.62 b	11.75 b	2.70 b	1.03 b
BAG24	23.24 b	0.70 b	14.87 c	10.12 b	2.81 b	1.28 a
BAG27	23.04 b	0.69 b	15.00 c	12.75 a	2.96 a	1.42 a
BAG38	22.24 c	0.76 b	13.87 c	11.50 b	2.80 b	1.17 b
BRS1216	30.42 a	0.73 b	16.87 b	12.75 a	2.87 b	0.92 b
BRS2299	25.93 a	0.72 b	13.25 c	11.62 b	2.44 b	1.03 b
BRS2336	23.66 b	0.77 a	13.62 c	12.37 b	2.49 b	0.63 b
BRS2357	25.13 a	0.79 a	15.00 c	13.25 a	3.09 a	0.99 b
BRS2314	17.62 c	0.78 a	15.00 c	11.25 b	2.77 b	1.14 b
BRS3213	19.26 c	0.88 a	13.37 c	11.75 b	2.77 b	0.78 b

Means followed by the same letter do not differ according to the Scott–Knott test at the 5% probability level.

**Table 3:** Mean micronutrient concentrations of the 64 clones evaluated in the clonal competition trial conducted in the municipality of Ouro Preto do Oeste, Rondônia, Brazil.

Clone	Boron	Copper	Iron	Manganese	Zinc	Sodium	Aluminum
AR106	77.61 a	6.75 b	235.00 a	145.00 a	8.75 b	73.25 a	237.50 a
R152	85.01 a	7.50 a	217.50 a	137.50 a	11.50 b	66.50 a	210.00 b
BG180	84.54 a	8.50 a	240.00 a	150.00 a	9.75 b	69.75 a	225.00 b
P42	73.84 b	6.00 b	225.00 a	107.50 b	11.25 b	37.75 b	230.00 b
CA1	84.26 a	5.50 c	222.50 a	137.50 a	11.00 b	42.00 b	297.50 a
RMD	75.09 b	5.75 c	190.00 b	127.50 b	10.75 b	37.00 b	232.50 b
GB7	76.68 a	6.50 b	182.50 b	145.00 a	11.25 b	50.75 a	230.00 b
R22	74.55 b	5.25 c	190.00 b	145.00 a	10.00 b	50.75 a	225.00 b
S05	80.45 a	5.75 c	185.00 b	140.00 a	10.25 b	53.25 a	245.00 a
WP6	73.26 b	6.00 b	205.00 a	145.00 a	10.50 b	55.50 a	250.00 a
L130	72.86 b	5.50 c	160.00 b	150.00 a	11.25 b	59.75 a	222.50 b
L1	72.99 b	5.00 c	190.00 b	145.00 a	10.25 b	56.25 a	230.00 b
GB1	70.64 b	5.25 c	210.00 a	142.50 a	11.75 b	59.25 a	245.00 a
GB4	80.17 a	5.25 c	222.50 a	155.00 a	10.75 b	63.25 a	240.00 a
AGL1	74.93 b	4.25 c	195.00 b	140.00 a	9.25 b	63.5 a	215.00 b
RE11	71.64 b	4.50 c	150.00 b	140.00 a	10.25 b	67.50 a	205.00 b
RE90	73.73 b	3.75 c	165.00 b	110.00 b	11.00 b	52.00 a	205.00 b
SK04	77.09 a	6.25 b	217.50 a	152.50 a	10.50 b	70.75 a	205.00 b
SK41	69.12 b	6.50 b	180.00 b	155.00 a	10.25 b	65.25 a	165.00 b
SK80	64.13 b	6.25 b	142.50 b	145.00 a	11.50 b	54.50 a	192.50 b
SK88	66.83 b	6.75 b	132.50 b	157.50 a	10.50 b	60.00 a	207.50 b
SK244	65.39 b	6.25 b	172.50 b	150.00 a	10.00 b	59.50 a	207.50 b
VP156	66.43 b	6.25 b	175.00 b	152.50 a	12.25 b	60.50 a	225.00 b
VP148	62.05 b	7.75 a	182.50 b	117.50 b	12.75 b	36.50 b	222.50 b
P50	70.31 b	7.25 a	170.00 b	110.00 b	9.75 b	34.50 b	250.00 a
Repiado	78.22 a	5.50 c	192.50 b	112.50 b	9.75 b	27.75 b	262.50 a
AS1	77.43 a	5.25 c	220.00 a	117.50 b	10.25 b	27.00 b	250.00 a
AS2	80.11 a	5.50 c	232.50 a	117.50 b	10.00 b	26.75 b	262.50 a
AS5	89.92 a	5.00 c	227.50 a	117.50 b	10.50 b	28.50 b	275.00 a
AS7	70.57 b	5.50 c	225.00 a	120.00 b	11.50 b	28.25 b	257.50 a
AS10	77.89 a	7.50 a	212.50 a	115.00 b	11.00 b	38.00 b	262.50 a
AS12	81.23 a	6.25 b	217.50 a	115.00 b	17.50 b	32.50 b	275.00 a
N13	81.57 a	6.25 b	237.50 a	115.00 b	11.25 b	26.25 b	262.50 a
N14	92.42 a	6.25 b	217.50 a	122.50 b	11.00 b	25.75 b	290.00 a
N16	83.02 a	5.25 c	215.00 a	122.50 b	9.75 b	29.50 b	270.00 a
N6	83.07 a	3.75 c	200.00 b	122.50 b	9.25 b	35.75 b	277.50 a
N8G8	85.13 a	4.75 c	232.50 a	125.00 b	9.75 b	39.25 b	302.50 a
RE22	92.64 a	5.00 c	190.00 b	107.50 b	9.00 b	30.25 b	270.00 a
GJ5	77.03 a	5.50 c	202.50 a	110.00 b	9.75 b	33.75 b	272.50 a
GJ8	74.73 b	4.75 c	210.00 a	107.50 b	9.00 b	28.25 b	220.00 b
GJ25	71.93 b	5.25 c	215.00 a	100.00 b	10.00 b	27.00 b	225.00 b
GJ2	82.07 a	4.25 c	197.50 b	100.00 b	10.50 b	33.00 b	210.00 b
GJ20	75.78 b	5.00 c	225.00 a	105.00 b	10.00 b	33.75 b	220.00 b
GJ30	83.62 a	5.00 c	200.00 b	107.50 b	11.00 b	23.00 b	212.50 b
LB10	82.36 a	5.25 c	217.50 a	97.50 b	9.75 b	35.50 b	210.00 b
LB15	84.81 a	5.00 c	232.50 a	90.00 b	8.75 b	35.25 b	227.50 b
LB30	86.17 a	4.75 c	220.00 a	105.00 b	7.75 b	34.00 b	205.00 b
LB33	79.29 a	7.50 a	192.50 b	140.00 a	110.00 a	74.50 a	182.50 b
LB68	72.05 b	6.50 b	232.50 a	140.00 a	9.50 b	55.25 a	205.00 b
LB80	86.38 a	5.00 c	217.50 a	110.00 b	9.00 b	59.25 a	215.00 b
LB88	80.91 a	3.25 c	247.50 a	115.00 b	9.75 b	51.50 a	207.50 b
LB101	87.01 a	6.25 b	225.00 a	107.50 b	8.75 b	47.75 a	217.50 b
BAG15	80.32 a	7.00 b	192.50 b	110.00 b	33.25 b	50.00 a	220.00 b
BAG21	83.43 a	4.50 c	195.00 b	110.00 b	8.50 b	44.25 b	210.00 b
BAG22	81.19 a	4.50 c	177.50 b	107.50 b	9.00 b	36.25 b	200.00 b
BAG24	77.03 a	4.75 c	215.00 a	112.50 b	9.00 b	50.50 a	197.50 b
BAG27	80.51 a	4.25 c	175.00 b	107.50 b	9.00 b	49.00 a	202.50 b
BAG38	71.71 b	6.00 b	195.00 b	112.50 b	9.25 b	46.25 a	192.50 b
BRS1216	65.88 b	6.75 b	180.00 b	112.50 b	13.00 b	41.25 b	182.50 b
BRS2299	78.46 a	6.25 b	140.00 b	110.00 b	11.00 b	48.00 a	177.50 b
BRS2336	68.09 b	8.75 a	185.00 b	115.00 b	10.75 b	44.50 b	180.00 b
BRS2357	71.86 b	7.25 a	200.00 b	122.50 b	12.25 b	48.50 a	182.50 b
BRS2314	68.14 b	8.00 a	197.50 b	112.50 b	8.75 b	47.75 a	182.50 b
BRS3213	71.19 b	6.75 b	227.50 a	137.5 a	11.50 b	70.75 a	197.50 b

Means followed by the same letter do not differ according to the Scott–Knott test at the 5% probability level.

**Table 4:** Chemical properties of a dystrophic Yellow–Red Oxisol from the Embrapa Rondônia Experimental Field, located in Ouro Preto do Oeste, Rondônia, Brazil, in 2021.

Soil Layer	pH	P	K	Ca^2+^	Mg^2+^	H + Al^3+^	Al^3+^	CEC	OM	m	V
(cm)	H_2_ O	mg dm^−3^	------------------ cmol_c_ dm^−3^ --------------						g kg^−1^	----- % ----	
0–10	5.7	31	0.37	4.14	1.17	3.96	0.0	9.64	15.7	0.0	59
10–20	5.7	11	0.26	3.19	0.75	2.61	0.0	6.81	7.1	0.0	62

CEC = cation exchange capacity; OM = organic matter; m = aluminum saturation index; V = base saturation percentage. Soil chemical analyses were performed before the establishment of the experimental field.

**Table 5:** Identification of the clones evaluated in the Rondônia State Clonal Competition Network Trial, conducted in the municipality of Ouro Preto do Oeste during the 2023–2024 growing season.

No	Clone	Origin	Status	No	Clone	Origin	Status
1	AR106	Aldnei Raasch ^3^	Public domain	33	AS1	Ademar Schmidt ^1^	Public domain
2	R152	Ronaldo Oliveira ^1^	Public domain	34	AS2	Ademar Schmidt ^1^	Public domain
3	BG180	Adilson Berger ^2^	Public domain	35	AS5	Ademar Schmidt ^1^	Public domain
4	P42	Wanderly Bernabé ^6^	Public domain	36	AS7	Ademar Schmidt ^1^	Public domain
5	CA1	Carlos Silva ^7^	Public domain	37	AS10	Ademar Schmidt ^1^	Public domain
6	RMD	Fredson Nunes ^3^	Public domain	38	AS12	Ademar Schmidt ^1^	Public domain
7	GB7	Gilberto Boone ^1^	Public domain	39	GJ5	Geraldo Jacomin ^4^	Public domain
8	R22	Ronaldo Vitoriano ^4^	Public domain	40	GJ8	Geraldo Jacomin ^4^	Public domain
9	S05	Sirley de Riz ^5^	Public domain	41	GJ25	Geraldo Jacomin ^4^	Public domain
10	WP6	Wanderley Peter ^5^	Public domain	42	GJ2	Geraldo Jacomin ^4^	Public domain
11	L130	Alcides Rosa ^2^	Public domain	43	GJ20	Geraldo Jacomin ^4^	Public domain
12	L1	Alcides Rosa ^2^	Public domain	44	GJ30	Geraldo Jacomin ^4^	Public domain
13	GB1	Gilberto Boone ^1^	Public domain	45	LB10	Laerte Braun ^4^	Public domain
14	GB4	Gilberto Boone ^1^	Public domain	46	LB15	Laerte Braun ^4^	Public domain
15	AGL1	Romário Lopes ^7^	Public domain	47	LB30	Laerte Braun ^4^	Public domain
16	RE11	Romário Lopes ^7^	Public domain	48	LB33	Laerte Braun ^4^	Public domain
17	RE22	Romário Lopes ^7^	Public domain	49	LB68	Laerte Braun ^4^	Public domain
18	RE90	Romário Lopes ^7^	Public domain	50	LB80	Laerte Braun ^4^	Public domain
19	SK04	Sérgio Kalk ^5^	Public domain	51	LB88	Laerte Braun ^4^	Public domain
20	SK41	Sérgio Kalk ^5^	Public domain	52	LB101	Laerte Braun ^4^	Public domain
21	SK80	Sérgio Kalk ^5^	Public domain	53	BAG15	Embrapa ^3^	Breeding
22	SK88	Sérgio Kalk ^5^	Public domain	54	BAG21	Embrapa ^3^	Breeding
23	SK244	Sérgio Kalk ^5^	Public domain	55	BAG22	Embrapa ^3^	Breeding
24	VP156	Valdecir Piske ^1^	Public domain	56	BAG24	Embrapa ^3^	Breeding
25	VP148	Valdecir Piske ^1^	Public domain	57	BAG27	Embrapa ^3^	Breeding
26	P50	Valdecir Piske ^1^	Public domain	58	BAG38	Embrapa ^3^	Breeding
27	Repiado	Valdecir Piske ^1^	Public domain	59	BRS1216	Embrapa ^3^	Cultivar
28	N13	Nivaldo Ferreira ^5^	Public domain	60	BRS2299	Embrapa ^3^	Cultivar
29	N14	Nivaldo Ferreira ^5^	Public domain	61	BRS2336	Embrapa ^3^	Cultivar
30	N16	Nivaldo Ferreira ^5^	Public domain	62	BRS2357	Embrapa ^3^	Cultivar
31	N6	Nivaldo Ferreira ^5^	Public domain	63	BRS2314	Embrapa ^3^	Cultivar
32	N8G8	Nivaldo Ferreira ^5^	Public domain	64	BRS3213	Embrapa ^3^	Cultivar

^1^ Alta Floresta do Oeste, Rondônia, Brazil; ^2^ Rolim de Moura, Rondônia, Brazil; ^3^ Ouro Preto do Oeste, Rondônia, Brazil; ^4^ Nova Brasilândia do Oeste, Rondônia, Brazil; ^5^ Cacoal, Rondônia, Brazil; ^6^ Alto Alegre dos Parecis, Rondônia, Brazil, ^7^ Novo Horizonte do Oeste, Rondônia, Brazil.

## Data Availability

The data supporting the findings of this study are presented in the article, and the plot-level data used in the statistical analyses are available from the corresponding author upon reasonable request.

## Abbreviations

BAG	Active Germplasm Bank
BRS	Cultivar Identification Prefix Used by Embrapa
CVe	Experimental Coefficient of Variation
CVg/CVe	Ratio between Genetic and Experimental Coefficients of Variation
Embrapa	Brazilian Agricultural Research Corporation
PCA	Principal Component Analysis
PC1	First Principal Component
PC2	Second Principal Component
